# Editorial: Mapping enzyme activity: from novel diagnostics to target-based therapeutics, how activity-based probes are improving our understanding of biological catalysts

**DOI:** 10.3389/fphar.2023.1271247

**Published:** 2023-08-22

**Authors:** André Luiz Lourenço

**Affiliations:** Department of Pharmaceutical Chemistry, School of Pharmacy, University of California, San Francisco, San Francisco, CA, United States

**Keywords:** activity-based probes, enzyme activity, functional proteomics, functional imaging, enzyme biomarkers

Enzymes are vital biological entities that enable living organisms to fulfill a fundamental condition for life: the efficient and selective catalysis of chemical reactions. Unsurprisingly, enzymes offer unique opportunities to improve our understanding of physiological systems and the pathogenesis of challenging human diseases. Every disease process is governed by a particular set of biochemical and molecular mechanisms featuring enzymes, whose activity may lead to the onset of a pathological state. Activity-based probes are highly selective active-site targeted chemicals used to label and monitor the activity state of an enzyme. The clever manipulation of activity-based probes can reveal complex physiological or pathological enzyme-substrate interactions. It can provide critical information on the cellular localization of enzymes, the extent of their expression and function, and foster the annotation of new enzyme drug targets.

With multilayered post-translational activity regulation occurring during disease onset, significant differences between enzyme abundance and activity are observed, making the precise mapping of enzyme activity across multiple conditions a critical unmet need.

This Research Topic aimed to navigate the most recent concepts and technologies to address this unmet need by exploiting enzyme activity to uncover novel pharmacological approaches in human health. We expect this collective of papers will be able to revisit the vast importance of hydrolases in the field and highlight the novelty presented by activity-based probes targeting oxidoreductases.

Activity-based probes were first described in 1990 and have become notorious in recent years, where many efforts have been carried out to improve our understanding of protein expression and function in biological systems. The field has advanced the initial goals of the technology, which aimed for the development of inhibitors for a particular target enzyme, towards the usage of activity-based probes to visualize and characterize enzyme activity within a complex proteome (Fang et al., 2021; Ramos-Llorca et al., 2022).

Historically, hydrolases such as proteases have been at the forefront development of activity-based probes as pharmaceuticals entities and novel theranostics (Sotiropoulou et al., 2022; Ćwilichowska et al., 2022). Most recently, the rapid use of activity-based probes was able to determine the complete substrate specificity profile of SARS-CoV-2 proteases, providing a valuable basis for the design of chemical compounds for potential early diagnosis and therapy of COVID-19 at a time when no vaccines or treatment were approved (Mahoney et al., 2021; Rut et al., 2021). Despite the challenges presented by COVID-19, recent years were also marked by advances in oncology. Zhao et al. (2021) developed a specific activity-based probe designed to detect extracellular granzyme B as it is secreted by activated immune cells in the tumor microenvironment. Pre-clinical data showed how this probe holds the potential to aid cancer therapy by highlighting tumors that will regress on treatment with immunomodulatory therapies and is entering its first-in-human phase I/II imaging study, with estimated completion in 2027 (Aggarwal, 2023).

Caspases represent a family of proteases that have been drawing attention as a target for activity-based probes due to their central role in apoptosis, inflammation, and fibrosis. The development of Emericasan (IDN-6556) has brought us evidence of how a novel activity-based pharmaceutical could achieve rapid clinical translation as a first-in-class, orally active, irreversible, pan-caspase protease inhibitor for the treatment of hepatic diseases such as nonalcoholic steatohepatitis and portal hypertension (Garcia-Tsao et al., 2019). Avrutsky and Troy assembles a collective of clinical reports of caspase-9 genetics, signaling, and cellular localization in human tissues. It also identifies gaps between experimental and clinical studies on caspase-9 and presents opportunities for further investigations to examine the consequences of caspase activity in human disease.

Proteases share the spotlight for activity-based probes development with another class of relevant targets among hydrolases—Lipases. Deng et al. and his associates have discussed the role of monoacylglycerol lipase in regulating the levels of the endocannabinoid 2-arachidonoylglycerol in the brain. Targeting this hydrolase may contribute to managing pathophysiology processes, such as inflammation and pain, bringing positive therapeutical outcomes to chronic diseases such as cancer and neurodegenerative diseases. Using a new substrate-based fluorescent assay in combination with activity-based protein profiling as an orthogonal approach, the authors were capable of screening a focused library of 320 organic compounds that led to the identification of cryptotanshinone and β-carbolines as potent and selective MAGL inhibitors showing antiproliferative activities against multiple cancer cells.

Activity-based probes extend their reach of application to other enzyme families beyond hydrolases. Abu Ghosh et al. briefly review the activity of CYP2C9 and shed light on its differential profile between Ethiopian versus Non-Ethiopian Jews. This microsomal cytochrome P450 oxidoreductase characterizes as one of the major catalytic enzymes responsible for the metabolism of frequently prescribed drugs, including NSAIDs, coumarin anticoagulants, angiotensin receptor antagonists, antidiabetic drugs, and the anticonvulsant phenytoin. The authors used phenytoin to determine the differential activity of CYP2C9 in Ethiopian versus Non-Ethiopian Jews utilizing a cohort of 300 healthy individuals. The authors correlated enzyme activity data with a panel of CYP2C9 genetic polymorphisms, characterizing a particular genotype (prevalent in Ethiopian Jews) that may hinder patients more susceptible to experiencing gastrointestinal bleeding complications during treatment with NSAIDs due to the differential activity of CYP2C9.

Oxidoreductases are also crucial enzymes for the ecological success of *Clostridioides difficile*. In most cases, gut commensal clostridia are opportunistic pathogens, relying on a unique, complex, and adaptable energy metabolism for their ecological success. Bustin et al. have used an activity-based protein profile approach to uncover proline reductase as a biomarker and metabolic driver for toxigenic *Clostridioides difficile* infection. Their paper sheds light on the role of Stickland reductases in Clostridia-specific amino acid fermentation and how their activity can be targeted with activity-based hydrazine probes as active site-directed inhibitors. Such an approach reveals the enzymatic activity of proline reductase, via its druggable cofactor modality, as a promising therapeutic target that would allow repopulation of direct *C. difficile* competitors for proline in the infection environment and restore colonization resistance in the gut.
